# *Arabidopsis thaliana* Plants’ Overexpression of the MYB Transcription Factor *VhMYB60* in the Face of Stress Hazards Enhances Salt and Cold Tolerance

**DOI:** 10.3390/ijms26041695

**Published:** 2025-02-17

**Authors:** Zhe Chen, Jinghan Wang, Wenhui Li, Xiang Chen, Changjia Zhao, Yanbo Guo, Yingnan Li, Zhuo Chen, Xingguo Li, Deguo Han

**Affiliations:** Key Laboratory of Biology and Genetic Improvement of Horticultural Crops (Northeast Region), Ministry of Agriculture and Rural Affairs, National-Local Joint Engineering Research Center for Development and Utilization of Small Fruits in Cold Regions, College of Horticulture & Landscape Architecture, Northeast Agricultural University, Harbin 150030, China; zhechen0313@163.com (Z.C.); wangjinghan041218@163.com (J.W.); wenhuili@neau.edu.cn (W.L.); cx01160202@163.com (X.C.); z772583jia@163.com (C.Z.); 15655436568@163.com (Y.G.); liyingnan1031@163.com (Y.L.); a02220019@neau.edu.cn (Z.C.)

**Keywords:** grapes, *VhMYB60*, salinity and chilly stress, transcriptional regulation, *Arabidopsis thaliana*

## Abstract

‘Beta’ (*Vitisriparia* × *V. labrusca*) is a vine fruit tree of the genus Vitis which is a cross between American and riparian grapes. In the current situation of grape production in northern regions, cold, drought, and salinity are important bottlenecks restricting its development, while some grape rootstocks with excellent traits show the disadvantage of poor resilience. ‘Beta’ (*Vitis riparia* × *V. labrusca*), one of the most extensively utilized rootstocks in viticulture, has demonstrated remarkable resilience to adverse conditions. However, the mechanisms by which ‘Beta’ rootstocks resist abiotic stresses are unknown and need to be further investigated. In this study, we successfully isolated and cloned a novel MYB transcription factor, *VhMYB60*, from the ‘Beta’ grapevine. This factor spans 972 base pairs and encodes a protein comprising 323 amino acids. Subcellular localization studies revealed that *VhMYB60* is predominantly expressed within the nucleus. Furthermore, tissue-specific expression analysis demonstrated that *VhMYB60* is more abundantly expressed in the mature leaves and roots of the grape plant. Further studies showed that salt and cold stress notably increased *VhMYB60* gene expression in both mature leaves and grape roots. Compared with the control, *Arabidopsis thaliana* (*Arabidopsis*) plants molecularly modified to overexpress *VhMYB60* exhibited enhanced salt and cold resistance and improved survival rates. Moreover, notable changes were detected in chlorophyll, malondialdehyde (MDA), proline, peroxidase (POD), catalase (CAT), and superoxide dismutase (SOD) levels. Concurrently, the expression levels of structural genes that are positively correlated with resistance to adversity stress were markedly elevated in *Arabidopsis* plants that overexpress *VhMYB60*. Consequently, *VhMYB60* may serve as a pivotal transcription factor in the regulation of ‘Beta’ resistance.

## 1. Introduction

In some northern areas in China, the extreme environmental conditions severely impact plant growth and development., among which salt and cold stress pose the greatest threat to fruit yield [1,2]. When plants are subjected to salt stress and cold stress, the metabolic level of free radicals in the cell body changes, the structure of the cell membrane is damaged, and the function of the cell membrane selective permeability fails, leading to osmotic pressure imbalance, ultimately causing rupture [3,4]. In order to maintain normal growth and development, plants spontaneously produce some changes in metabolic level to achieve the purpose of resisting stress. In this whole process, transcription factors play a huge role [5]. MYB transcription factors represent a widely recognized family of transcriptional regulators. MYB transcription factors represent a widely recognized family of transcriptional regulators [6]. The most fundamental difference between it and other transcription factor families is that it has a unique MYB domain [7].

Usually, the MYB domain consists of 50 amino acid fragments, which can be subdivided into three categories according to different structural characteristics: the R1 structural domain with the structure W-(X20)-W-(X20)-A, the R2 structural domain with the structure W-(X19)-W-(X19)-W, and the R3 structural domain with the structure F/I-(X18)-W-(X18)-W [8]. The MYB superfamily is categorized into four distinct subfamilies, determined by the number of neighboring repetitive and conserved structural domain sequences found within the MYB domains. These subfamilies are differentiated based on these specific sequence patterns., the R2R3-MYB transcription factors are the most studied subfamily with two highly conserved MYB structural domains, which are capable of specifically binding to DNA and modulating the expression of target genes [9]. Research indicates that the R2R3-MYB transcription factor is pivotal not only for the growth and development of plants but also for helping them cope with abiotic stresses [10,11,12,13,14]. This factor enables prompt regulatory actions that safeguard plants against challenging conditions like drought, cold spells, and salinity [15,16,17]. Among them, CgMYB1, an R2R3-MYB transcription factor, has been found in *Chenopodium glaucum*. Under stress from salt and cold weather, the expression levels of *CgMYB1* in this species saw a substantial increase. Moreover, when *CgMYB1* was overexpressed in the model organism *Arabidopsis*, it notably enhanced the plant’s resilience to both salt and cold stress [18]. *BpMYB95*, a transcription factor, is linked to the salt tolerance of *Betula platyphylla Suk*. When the birch tree is subjected to saline conditions, this factor can notably boost its salt tolerance and mitigate symptoms like leaf browning and wilting that are brought about by salt stress [19]. The PsMYB99 transcription factor in Siberian apricot was able to reduce the effect of low temperature on flowering during flowering in apricot trees, thereby protecting against loss of fruit yield [20]. *PbMYB1L*, discovered in *Pyrus bretschneideri*, boosts cold tolerance and promotes anthocyanin production in genetically modified *Arabidopsis* by influencing the expression of 14 genes linked to cold response and anthocyanin synthesis pathways [21].

‘Beta’ is a high-quality grape variety in the northern region of China, which possesses high quality resistance to adversity and can grow normally under the adverse environmental conditions of salinity and high temperature in the northern part of the country, and is therefore often used as grape rootstock material. Recent studies on *MYB60* have shown that this transcription factor plays a crucial role in enhancing the resistance of various plants to abiotic stresses such as drought, salt stress and low temperature [22,23,24,25,26]. However, in the current study, the specific role of *MYB60* in ‘Beta’ is still not fully explored. In view of the increasing importance of abiotic stress resistance in viticulture, we combined the published grape genome data to screen out the key differential gene *VhMYB60* from the grape abiotic stress transcriptome data and conducted further studies, aiming to increase the understanding of MYB transcription factors in grapes and provide valuable theoretical insights into its potential applications in molecular breeding.

## 2. Research Results

### 2.1. VhMYB60 Gene Cloning and Bioinformatic Analysis

**Figure 1:**
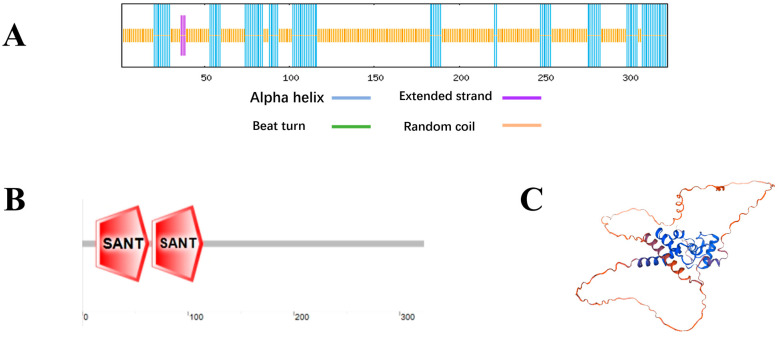
Structure of the *VhMYB60* protein. the (**A**) secondary structure, (**B**) functional domain, and (**C**) tertiary structure of *VhMYB60*.

The *VhMYB60* coding sequence (CDS), consisting of 323 amino acids and 972 bases (Figure S1), was assessed using ProtParam (https://web.expasy.org/, accessed 7 October 2023.). The *VhMYB60* protein had an estimated molecular weight of about 36.43 kDa and a calculated isoelectric point of 6.13. The amino acid composition of the *VhMYB60* protein showed notable concentrations of serine (Ser) (10.2%), leucine (Leu) (8.0%), glutamic acid (Glu) (6.8%), aspartic acid (Asp) (6.2%), and other amino acids. The aliphatic index of the *VhMYB60* protein was 65.85, which is below 100. Meanwhile, the average hydrophilicity coefficient was approximately −0.776, which is relatively negative. This combination indicates that the protein is more hydrophilic. The instability index (II) was 48, which is greater than 40, suggesting that the *VhMYB60* protein is unstable. The phylogenetic tree shows the evolutionary relationship of *VhMYB60* with other plants. As can be seen from the tree diagram, *VhMYB60* has the highest homology with *VrMYB60*, followed by *VaMYB60* and *VvMYB60B*( Figure S2). NPS (https://npsa.lyon.inserm.fr/cgi-bin/npsa_automat.pl?page=/NPSA/npsa_sopma.html, accessed 7 October 2023.) prediction reveals the *VhMYB60* protein secondary structure. The predicted results show that *VhMYB60* is composed of 29.41% α-helix, 0% β-turn, 0.93% extended chain, and 69.66% random coil (Figure 1A). *VhMYB60* contains two SANT domains located at residues 13-63aa and 66-114aa (Figure 1B). The tertiary structure predicted by SWISS-MODEL contains two HTH regions (Figure 1C), the same number as the predicted conserved domain. *VhMYB60* is therefore classified as an R2R3-MYB transcription factor among members of the MYB transcription factor family.

### 2.2. Subcellular Localisation of the VhMYB60 Gene in Tobacco Benjamina

**Figure 2:**
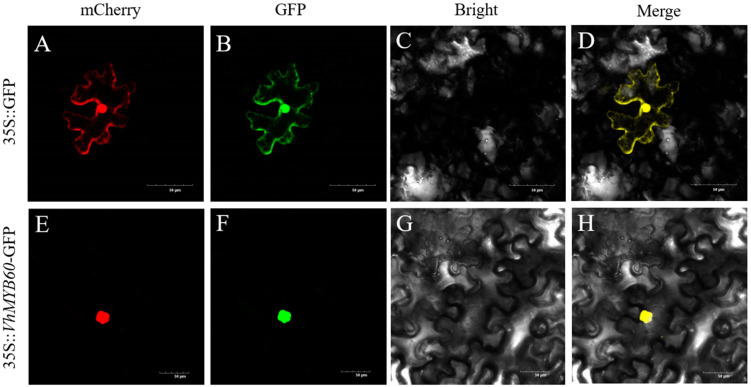
Subcellular localization of *VhMYB60* protein; 35S:: *VhMYB60*:: GFP was expressed transiently into tobacco leaves with 35S:: GFP as positive control. (**A**,**E**) mCherry; (**B**,**F**) GFP signals; (**C**,**G**) bright field; (**D**,**H**) merge. mCherry as a nuclear marker. Yellow indicates GFP and mCherry colocalization. Scale bars correspond to 50 um.

To identify the subcellular distribution and functional significance of *VhMYB60* as a transcriptional regulator in plant cells, We engineered the fusion vector *VhMYB60*-pcambia1300 and introduced it into the leaves of *Nicotiana benthamiana* through transient transformation. Using laser confocal microscopy, we then analyzed the fluorescence distribution within the tobacco leaves to assess the results. The results showed that *VhMYB60* was located in the nucleus of tobacco epidermal cells (Figure 2).

### 2.3. Analysis of the Expression Level of VhMYB60 in ‘Beta’

**Figure 3:**
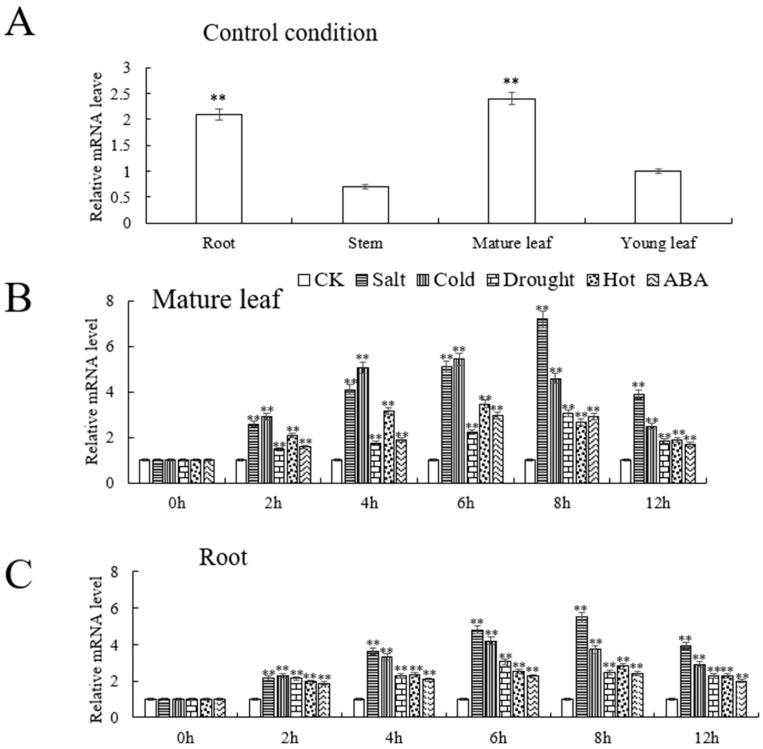
Expression pattern of *VhMYB60* Induced by abiotic stress in different parts of ‘Beta’ (**A**) Expression level of *VhMYB60* in four different parts of ‘Beta’ grown under normal culture conditions. (**B**,**C**) *VhMYB60* expression in ‘Beta’ mature leaves and roots after 0–12 h of salt, cold, drought, high temperature, and ABA stress treatments ** *p*-value ≤ 0.01.

We analyzed the expression of *VhMYB60* in different parts of ‘Beta’ plants using cDNA and performed RT-qPCR, and observed that compared with other plant tissues, the highest expression of *VhMYB60* was found in mature leaves of ‘Beta’, followed by roots, In contrast, the lowest expression levels of *VhMYB60* were observed in young leaves and stems (Figure 3A). These findings suggest that *VhMYB60* is regulated by tissue-specific regulation, with mature leaves being the main site of expression.

After subjecting the samples to stress treatment using 100 mM NaCl, 6% PEG6000, temperatures of 4 °C and 37 °C, as well as 100 µL ABA for a duration of 12 h, the expression levels of *VhMYB60* demonstrated varying degrees of elevation in comparison to the control group. Notably, the most pronounced alterations were observed in both mature leaves and roots, where the expression of *VhMYB60* notably surged at the 8 h mark of the stress treatment with 100 mM NaCl. *VhMYB60* expression peaked at 7.1-fold and 5.5-fold of the control, respectively (Figure 3B,C). In mature leaves and roots, the expression of *VhMYB60* reached its peak 6 h after cold stress treatment. In mature leaves, the level was 5.4-fold higher than the control, and in roots, it was 4.2-fold higher than the control (Figure 3B,C). The findings showed that salt and cold exposure markedly increased *VhMYB60* expression in mature leaves and roots under various abiotic stress conditions.

### 2.4. Overexpression of VhMYB60 Significantly Enhances Salt Tolerance in Arabidopsis

**Figure 4:**
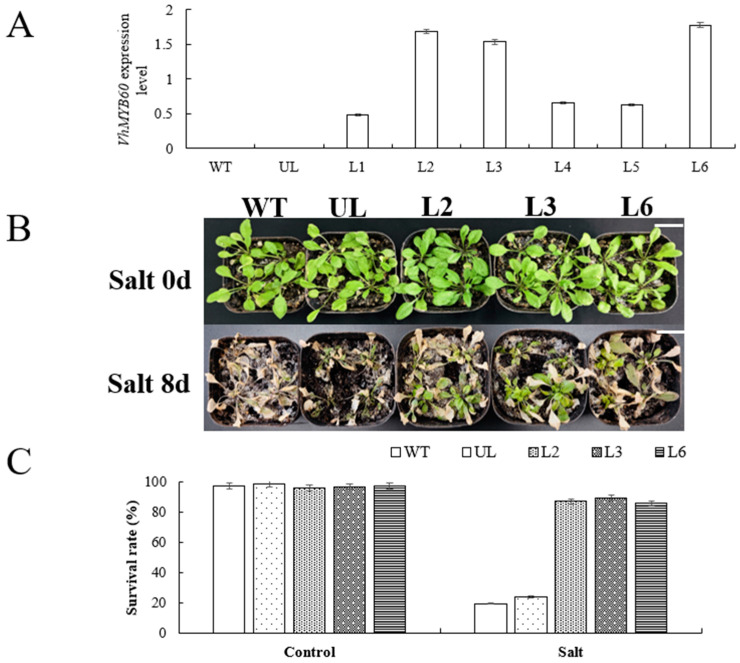
Overexpression of *VhMYB60* enhances salt tolerance in *Arabidopsis*. (**A**) Comparison of *VhMYB60* expression in *Arabidopsis* grown under normal culture conditions in WT, UL, and transgenic lines L1-L6. (**B**) Phenotype maps of WT, UL, L2, L3, and L6 after salt stress treatment (salt stress for 0 d and salt stress for 8 d). (**C**) Comparison of survival rates of WT, UL, L2, L3, and L6 after salt stress treatment. Applying WT as a guide. An error bar (*n* = 3) represents the standard deviation.

**Figure 5:**
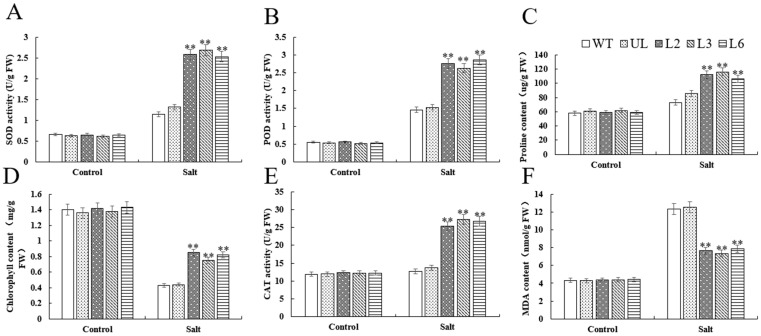
Related physiological indexes of *Arabidopsis* under salt stress after overexpression of *VhMYB60*. Analysis of (**A**) SOD, (**B**) POD, (**C**) proline, (**D**) chlorophyll, (**E**) CAT, (**F**) MDA in *Arabidopsis* at different salt stages (salt 0 d and salt 8 d), using WT indicators as controls. The SD is represented by an error bar (*n* = 3). (**) *p*-value ≤ 0.01.

To explore the function of *VhMYB60* in plants under salt and cold stress conditions, we conducted a screening of Arabidopsis and unloaded control (UL) plants overexpressing *VhMYB60* in the T_1_ generation for stress tolerance. We measured the expression levels of *VhMYB60* in the T_2_ generation by RT-qPCR, using wild-type (WT) and UL plants as controls. (Figure 4A). The findings revealed that *VhMYB60* expression was absent in WT and UL plants but was present at varying levels in transgenic lines (L1–L6), confirming successful integration and expression of *VhMYB60* in *Arabidopsis*. From these lines, three (L2, L3, and L6) exhibiting higher expression levels were chosen for further cultivation, ultimately yielding homozygous T_3_ generation transgenic *Arabidopsis* lines. To investigate the performance of *Arabidopsis* after the overexpression of *VhMYB60* under high salt stress, salt stress treatment experiments were performed on the screened (L2, L3, and L6) ULs as well as WTs. Under standard conditions, the phenotypes of the transgenic plants (L2, L3, and L6) were found to be comparable to those of the UL and WT seedlings (Figure 4B). Following 8 days of salt stress exposure, the leaves of transgenic plants (L2, L3, and L6), UL, and WT plants exhibited notable discoloration, with some displaying a grayish and purplish hue, particularly in the UL and WT plants (Figure 4B). In standard conditions, the survival rates of all lines were found to be comparable. Upon returning to normal culture conditions after salt stress, the survival rates for overexpression lines (L2, L3, and L6) were recorded at 86.9%, 89.2%, and 85.6%, respectively. In contrast, the survival rates for UL and WT plants were 19.5% and 23.8%, respectively. (Figure 4C). The observed discrepancy provides clear evidence that *Arabidopsis* plants overexpressing *VhMYB60* demonstrate enhanced resistance to salt stress and exhibit a higher survival rate in high-salinity conditions compared to UL and WT. In addition to the phenotypic observation, a series of physiological indexes related to stress resistance were measured on transgenic *Arabidopsis* after salt stress, and the data showed that there was no significant difference between L2, L3, L6, and the control groups (WT and UL) under normal culture conditions. After being subjected to salt stress treatment, *Arabidopsis* plants with overexpression of *VhMYB60* exhibited notable rises in the levels of three antioxidant enzymes (superoxide dismutase, peroxidase, and catalase) when compared to the control groups (wild type and untransformed lines). This suggests that these transgenic *Arabidopsis* possess more robust antioxidant capabilities. There was a marked increase in the content of PRO, which serves as an osmoregulatory substance, and it would effectively alleviate plant dehydration induced by salt stress. Meanwhile, chlorophyll content and MDA levels showed significant differences between the overexpression and control groups. (Figure 5A–F) The results of the studies indicated that overexpressing *VhMYB60* significantly improves the salt stress resistance of *Arabidopsis*, suggesting its vital role in helping the plant adapt to saline conditions.

### 2.5. VhMYB60 Activates Salt Stress-Related Genes

**Figure 6:**
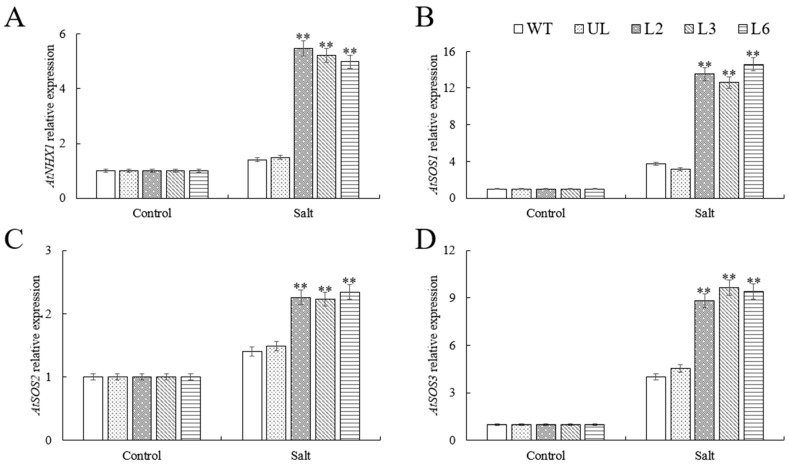
qRT-PCR was used to detect the expression of downstream genes related to salt stress resistance in WT, UL, and transgenic *Arabidopsis*. Relative expression levels of (**A**) *AtNHX1*, (**B**) *AtSOS1*, (**C**) *AtSOS2*, (**D**) *AtSOS3* at different salt stages (salt 0 d and salt 8 d). WT was used as control. Three repeated mean and standard deviation were taken as data. The standard deviation is represented by an error bar (*n* = 3). (**) *p* value ≤ 0.01.

To explore the regulatory function of *VhMYB60* genes under salt stress conditions, we focused on several pivotal genes associated with the salt stress response pathway, namely *AtSOS1*, *AtSOS2*, *AtSOS3*, and *AtNHX1*. These genes were analyzed using RT-qPCR, and the fluorescence quantification results revealed that, in the absence of stress, there were no substantial differences in the expression levels of these key genes between the overexpression strains (L2, L3, L6) and the control groups (UL and WT). However, following exposure to salt stress, a marked increase in the expression of these genes was observed in the overexpression lines (L2, L3, L6), significantly surpassing the levels noted in the WT and UL groups (Figure 6A–D). This strongly proved that *VhMYB60* activated the salt stress resistance pathway and enhanced the survival ability of *Arabidopsis* under salt stress.

### 2.6. Overexpression of VhMYB60 Significantly Enhances Cold Resistance in Arabidopsis

**Figure 7:**
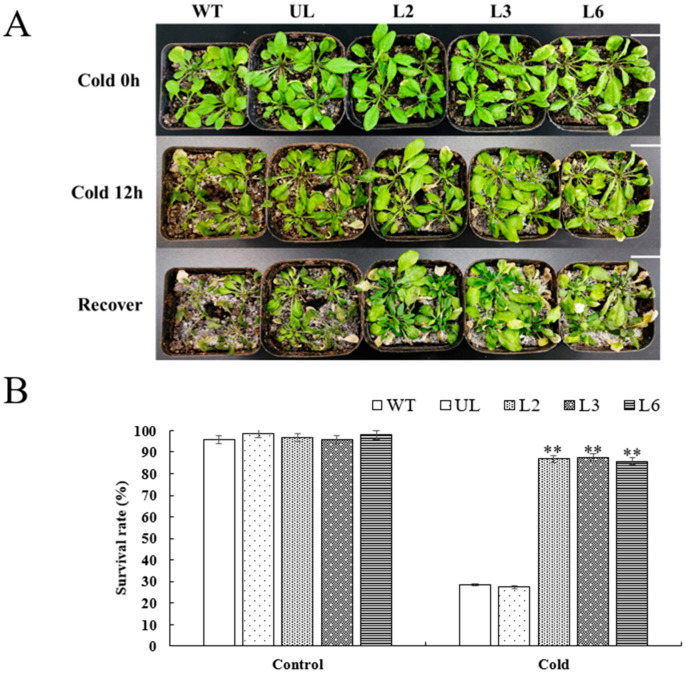
*Arabidopsis* phenotypic alterations and survival rate under cold stress. (**A**) The *Arabidopsis* phenotype (Cold 0 h, 12 h and recover), with a scale of 4 cm. (**B**) Survival rate of *Arabidopsis* under CK (cold 0 h) and cold stress (cold 12 h). The SD is represented by an error bar (n = 3). (**) *p* value ≤ 0.01.

Along with salt stress treatment, we also performed cold stress treatment on (L2, L3, L6) with WT and UL after overexpressing *VhMYB60*. There were no phenotypic differences among the strains under normal growth conditions in the early stage, but after 12 h of cold treatment at −4 °C, the overexpression strains (L2, L3, L6) and WT and UL showed some phenotypic changes, and the common features were that the leaf color of all strains became darker and shriveled, and the growth rate was significantly slowed down. The differences were that L2, L3, and L6 overexpressing *VhMYB60* showed less leaf wilting than WT and UL in the control group (Figure 7A). Seven days after the return to normal temperature, the control WT and UL did not show any improvement in the wilting condition, and even died, with survival rates of 28.5% and 27.4%, respectively (Figure 7B).

In contrast, after overexpression of *VhMYB60*, *Arabidopsis* (L2, L3, and L6) the growth rate slowed down at −4 °C. But gradually recovered on growth potential and had new leaf growth after normal culture conditions were restored. The survival rates were at 86.8%, 87.6%, and 85.8%, respectively (Figure 7B). This result serves as strong evidence that *VhMYB60* may improve the resistance of plants to cold stress.

**Figure 8:**
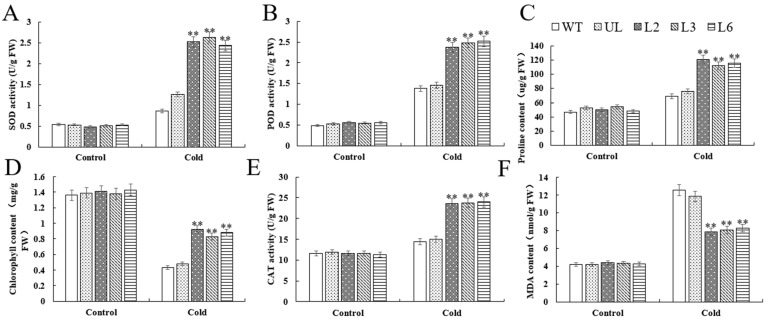
Impact of *VhMYB60* gene on *Arabidopsis* cold tolerance index. WT was utilized as the control in (**A**) SOD, (**B**) POD, (**C**) proline, (**D**) chlorophyll, (**E**) CAT, and (**F**) MDA. There is an error bar (*n* = 3) that shows the SD. *p* value (**) < 0.01.

To confirm *VhMYB60*’s role in enhancing cold stress tolerance, we assessed cold-related physiological indicators. The data showed that, under normal culture conditions, there was no significant difference in the expression of enzymatic activity between L2, L3, and L6 overexpression lines compared to the untransformed lines (UL) and wild-type (WT), and after exposure to cold stress, the overexpression lines exhibited a notable upsurge in the enzymatic activities of SOD, POD, and CAT. Concurrently, significant alterations were observed in the levels of proline, chlorophyll, and malondialdehyde (Figure 8A–D). In conclusion, the cold tolerance of *Arabidopsis* was significantly enhanced by the overexpression of *VhMYB60*.

### 2.7. VhMYB60 Activates Cold Stress-Related Genes

**Figure 9:**
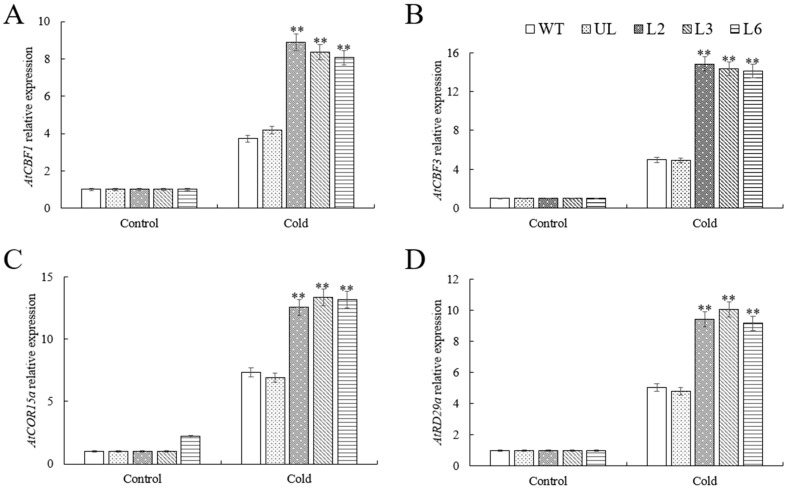
Expression levels of genes linked to cold stress in transgenic, UL, and WT *Arabidopsis* under cold stress conditions. (**A**) *AtCBF1*, (**B**) *AtCBF3*, (**C**) *AtCOR15a*, (**D**) *AtRD29a* relative expression levels. There is an error bar (*n* = 3) that shows the SD. *p* value (**) < 0.01.

To explore the role of *VhMYB60* in cold stress tolerance, we analyzed key CBF pathway genes (*AtCBF1*, *AtCBF3*, *AtCOR15a*, *AtRD29a*) using RT-qPCR. The study revealed that under normal growth conditions, the expression levels of key genes in the overexpression group were virtually indistinguishable from those in the control group. However, the tables turned after 12 h cold stress treatment. Specifically, the overexpression lines (L2, L3, L6) exhibited a marked upsurge in the expression of *AtCBF1*, *AtCBF3*, *AtCOR15a*, and *AtRD29a* compared to the control groups WT and UL (Figure 9A–D). This finding underscores the role of *VhMYB60* in activating the CBF signaling pathway, which plays a pivotal role in the plant’s cold stress response.

## 3. Discussion

The ‘Beta’ grape variety is commonly employed as rootstock material for grafting purposes [27]. While numerous studies have highlighted the role of MYB transcription factors in regulating responses to abiotic stress, the specific mechanisms governing this process in ‘Beta’ remain poorly understood. In this paper, we focused on the function of *VhMYB60* under abiotic stress, and through a series of bioinformatics analyses, we identified that *VhMYB60* belongs to the R2R3 type of MYB transcription factors, which possesses a special conserved structural domain (Figure 3B). At the same time, we cloned *VhMYB60* from ‘Beta’, constructed an overexpression vector, and transferred it into *Arabidopsis* plants with *Agrobacterium* as the mediator. We conducted a deeper investigation into how *Arabidopsis* responds to cold and salt stress following the overexpression of *VhMYB60*, examining both molecular and physiological aspects. Ultimately, our findings revealed that *VhMYB60* plays a crucial role in enhancing the plant’s ability to withstand these harsh conditions, effectively boosting its resistance to cold and salt stress.

Under adverse growth conditions, plant growth and development are hindered, and physiological markers linked to abiotic stress undergo changes [28,29,30,31]. In our research, we observed notable distinctions between the wild type (WT) and the UL of *Arabidopsis thaliana* following the overexpression of *VhMYB60* when subjected to both cold and salt stress, especially in comparison to the control group. First, in terms of enzyme activity related to abiotic stress resistance, SOD, POD, and CAT, are important antioxidant enzymes, and SOD is able to convert excessive intracellular superoxide anion into hydrogen peroxide and oxygen to avoid cellular damage [32,33,34]. POD can catalyze the reaction between hydrogen peroxide and organic matter to achieve the effect of scavenging ROS and generating organic matter and water [35,36,37]. CAT, on the other hand, is able to directly break down hydrogen peroxide to avoid damage caused by the excessive accumulation of hydrogen peroxide in cells [38,39]. The results of our experiments revealed that *Arabidopsis thaliana* plants overexpressing *VhMYB60* exhibited markedly elevated levels of SOD, POD, and CAT enzyme activities compared to both the wild-type (WT) and UL controls (Figure 8A–D). This finding strongly suggests that *VhMYB60* plays a pivotal role in enhancing the plant’s resilience to salt and cold stress by boosting the activity of these key enzymes. Additionally, proline, a well-known osmoprotectant, was observed to mitigate intracellular water loss in plants when exposed to harsh environmental conditions, further supporting the plant’s ability to withstand stress [40]. Under salt stress and cold stress treatments, high levels of proline will help maintain cellular stability, reduce ROS accumulation, and act as an antioxidant [41]. The data showed that *Arabidopsis*, after overexpression of *VhMYB60*, had a higher proline content relative to WT and UL, suggesting that *VhMYB60* may maintain a stable intracellular environment in plant cells by increasing the proline level of the plant in order to achieve the effect of avoiding cellular damage and increasing the resistance of the plant to adverse environments. Chlorophyll content is frequently utilized as an indicator of plant stress resistance, as it is essential for growth and energy metabolism. In the high salt and low temperature environment, the photosynthesis of plants is inhibited, which leads to the reduction in chlorophyll content. Therefore, chlorophyll levels serve as a key indicator for assessing plant resilience to salinity and low temperatures [42,43,44]. From our experimental results, after overexpression of *VhMYB60, Arabidopsis* was able to maintain a higher chlorophyll level under salt and cold stress treatments, relative to the control group, which also indicated that overexpression of *VhMYB60* enhanced *Arabidopsis* ability to withstand salt and cold stress. The content of MDA was highly correlated with the degree of oxidative damage to the plant cells [45,46,47], *Arabidopsis thaliana* overexpressing *VhMYB60* had lower MDA content relative to WT than UL, which implies that the overexpressing lines were less affected by salt and cold stress.

MYB transcription factors can boost plant stress tolerance by activating or modulating downstream gene expression [48,49,50]. To investigate how *VhMYB60* influences downstream genes under high salinity and cold stress, we gathered samples and analyzed the expression patterns of critical genes under two specific treatment scenarios. The findings revealed that *VhMYB60* plays a positive regulatory role in the expression of *AtSOS1*, *AtSOS2*, *AtSOS3*, and *AtNHX1* within the SOS pathway (Figure 8A–D). Furthermore, under low-temperature conditions, *VhMYB60* was shown to boost the transcript levels of *AtCBF1*, *AtCBF3*, *AtCOR15a*, and *AtRD29a* (Figure 9A–D).

**Figure 10:**
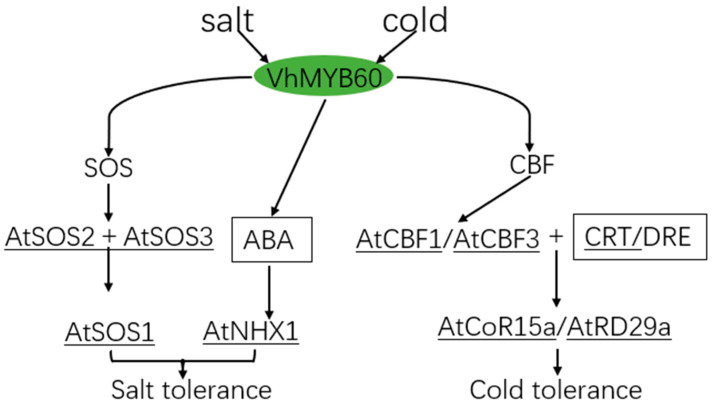
A potential mechanistic model of *VhMYB60* adaptation to cold and salt stress.

In conclusion, we anticipated the molecular model of *VhMYB60* in response to salt and cold stress (Figure 10). *VhMYB60* promotes downstream salt stress response genes under salt stress, and during this mechanism of action, *VhMYB60* activates *AtNHX1* to isolate sodium ions in the vesicle to achieve a reduction in the concentration of sodium ions. SOS3 senses a rise in the concentration of calcium ions in the cellular environment and activates the AtS0S2 gene in the process. *AtSOS2* then phosphorylates *AtSOS1*, which increases the activity of *AtSOS1* to achieve the purpose of regulating Na^+^/H^+^ to improve the plant’s tolerance to salt stress and to promote the excretion of sodium ions.

## 4. Materials and Methods

### 4.1. Plant Materials and Treatments

The shoot induction medium, composed of 4.3 g/L MS, 0.2 mg/L indole-3-butyric acid, and 0.5 mg/L 6-aminopurine, was used to culture histocultured seedlings of ‘Beta’ plants over a span of roughly 30 days [51]. For root induction, well-developed histocultured seedlings were transferred to a rooting medium containing 4.3 g/L MS, 0.2 mg/L IBA, and 0.1 mg/L 6-BA. The growing environment was maintained at 22 °C with 80% ± 5% humidity and a 16 h light/8 h dark cycle. Once the seedlings exhibited strong root growth, they were moved to a hydroculture system using Hoagland nutrient solution. The same cultivation conditions were applied to wild Columbia-type *Arabidopsis* seedlings. From the ‘beta’ hydroponic seedlings, 50 healthy plants with complete roots, stems, and leaves were selected for stress treatment according to the method of Han et al. [52]. After sampling, they were stored at −80 °C for further analysis

### 4.2. Isolation and Cloning of VhMYB60

Total RNA was extracted from the leaves of ‘Beta’ using the Total RNA Extraction Kit-Trizol Method (Wan Lei bio, Shenyang, China), and the LunaScript^®^ RT Master Mix Kit (NEB, Beijing, China) was subsequently used to reverse-transcribe the RNA into cDNA [53]. The total RNA was extracted from the leaves of ‘Beta’ and reverse-transcribed into CDNA using the LunaScript^®^ RT Master Mix Kit (Primer-free), the cDNA was amplified by polymerase chain reaction (PCR) using the *VvMYB60* nucleotide sequence in the genome assembly of grapevine (*Vitis vinifera* L.) (XM_034837254.1), and the cDNA was purified using the DNA product of Conway Century. PCR products were recovered using the Conway Century DNA product purification kit (KWbiio, Taizhou, China). The recovered products were used to ligate the T5 vector pEASY^®^-T5 Zero Cloning Kit, (Trans Gen Biotech, Beijing, China), and the grown single colonies were sent for sequencing, and the sequencing results were subjected to sequence comparison with DNAMAN5.2 and translated into amino acid sequences.

### 4.3. Subcellular Localisation of VhMYB60

Based on the sequence of the *VhMYB60* gene-coding region and the sequence characteristics of the pCAMBIA1300-GFP vector (Bioon, Shanghai, China), BamHI and KpnI were selected as restriction endonuclidene sites in this study, and specific primers containing the above enzyme restriction sites were designed for homologous recombination experiments (Table S1). The pCAMBIA1300-GFP vector was linearized by BamHI and SalI, and the double restriction site and homologous arm were linked to the target gene by PCR. Finally, the overexpression vector was constructed by homologous recombination [54]. Agrobacterium rhizogenes was ligated to both the empty vector and the successfully ligated *VhMYB60*-pCAMBIA1300-GFP expression vector. The successfully ligated Agrobacterium spp. were resuspended with resuspension solution, followed by shaking with a thermostatic shaker (180 rpm 28 °C) for 2–3 h. Then, the infiltration solution was injected with a syringe into 5–6-week-old Benjamin’s tobacco leaves, and incubated in dark culture for 24 h and in light culture for 48 h. The infiltration solution was then incubated with a laser confocal microscope and then in light culture for 48 h, and then in light culture for 24 h, and then in light culture for 48 h. This was followed by observation under a laser confocal microscope (LSM 900, Precise, Beijing, China).

### 4.4. Bioinformatics Analysis of VhMYB60

The nucleotide sequences of *VhMYB60* were translated into protein sequences using the NCBI database and DNAMAN 6 software. The ExPASy ProtParam tool was then used to analyze these protein sequences, enabling the prediction of the average hydrophilicity coefficient, theoretical isoelectric point (pI), and molecular weight of the *VhMYB60* protein [55]. Additionally, this analysis was facilitated by the ExPASy ProtParam tool, accessed on 7 October 2023. Predictions for the protein’s primary and secondary structures were performed with SOPMA. The VhMYB2 protein sequence was input into the SWISS-MODEL website for tertiary structure prediction. The translated amino acid sequences were analyzed using BLAST on NCBI (https://blast.ncbi.nlm.nih.gov/Blast.cgi, accessed 8 November 2023), and sequences with significant homology were chosen to be downloaded. Subsequently, a phylogenetic tree analysis was conducted using MEGA 7.0.

### 4.5. Expression Analysis of VhMYB60 Gene

Following the methodology outlined by Han et al. [56], RT-qPCR was employed to assess *VhMYB60* expression. Custom RT-qPCR primers were crafted by targeting the conserved sequences of the actin and *VhMYB60* genes (Table S1). For the RT-qPCR process, the SYBR^®^ Green Realtime PCR Master Mix, sourced from TaKaRa in Beijing, China, was employed to ensure accurate and reliable results. The relative expression of *VhMYB60* was determined using the 2-∆∆Ct method. The experiment was performed with three technical replicates.

### 4.6. Vector Preparation and Transformation of Arabidopsis Plants

The amplification primers were designed based on the CDS of the target gene. The pCAMBIA1300 plasmid, which contains the CaMV 35S promoter, was used to add the homology arm and double cleavage site (KpnI and BamHI) to the amplification primer sequence. Then, the *VhMYB60*-pCAMBIA1300 and the pCAMBIA1300 were added to the amplification primer sequence. Subsequently, the constructed 35S::*VhMYB60*-GFP vector plasmid was transferred into Agrobacterium rhizogenes and transformed *Arabidopsis* with Agrobacterium to obtain positive plants [57]. After 24 h of dark incubation followed by 48 h of normal light incubation (2–3 infestations), the seeds were harvested and T0 generation seeds were planted in the screening medium. The seedlings that had developed during the screening were then planted to harvest the seeds, and the transcripts were identified by extracting the RNA. Transgenic plants were seeded until the T3 generation, and the vigorous ones (L2, L3, L6) were chosen for further research after RNA was extracted from the seeds.

### 4.7. Analysis of Physiological Indicators After Stress Overexpression of VhMYB60 in Arabidopsis thaliana

The overexpression group (L2, L3, L6) and the control group (WT, UL) were cultured in a light incubator for 5 weeks (temperature 22 °C, humidity 85%, light/dark cycle 16 h/8 h). Salt stress treatment for 8 d and cold stress treatment for 12 h were performed, respectively. During the stress treatments, the condition of *Arabidopsis* was recorded and the leaves were collected after liquid nitrogen snap-freezing and refrigerated at −80 °C for subsequent experiments. MDA, chlorophyll (Ch), and proline were quantified using thiobarbituric acid, spectrophotometric and acid digestion methods, respectively [58]. Meanwhile, CAT, peroxidase (POD), and SOD activities were determined using UV spectrophotometry, the guaiacol method, and the tetranitrotetrazolium blue reduction method, respectively [59].

### 4.8. Analysis of Stress-Related Genes in Arabidopsis thaliana-Overexpressing VhMYB60

RNA was extracted from *Arabidopsis* leaves under normal culture conditions, salt stress, and cold stress treatments, respectively, and reverse-transcribed into cDNA. The resulting cDNA served as the template for subsequent RT-qPCR assays to analyze genes associated with the downstream targets of *VhMYB60*. Salt stress-related genes *(AtNHX1, AtSOS1, AtSOS2*, *AtSOS3*). Cold stress-related genes (*AtCBF1*, *AtCBF3*, *AtCOR15a*, *AtRD29a*). The downstream response genes and internal reference gene-specific primers were detailed in the attached table (Table S1).

### 4.9. Statistical Analyses

For each sample in this experiment, three biological replicates were conducted. Pertinent indicators were collected, processed, and analyzed. The experimental results were expressed as the mean ± standard error (SE) [60]. Statistically significant differences (* *p* < 0.05, ** *p* < 0.01) were determined using analysis of variance (ANOVA) performed with SPSS 26.0 software.

## 5. Conclusions

In summary, this study successfully isolated and identified the *VhMYB60* gene in grape rootstock ‘Beta’ (Vitis labrusca × Vitis riparia), and confirmed the close relationship between *VhMYB60* and *VrMYB60* through amino acid sequence analysis. Subcellular localization experiments showed that *VhMYB60* was mainly localized in the nucleus. The gene was highly expressed in different tissues such as mature leaves and roots, and was significantly responsive to high salt and low temperature signals. When *VhMYB60* is overexpressed in *Arabidopsis*, the physiological indicators of the plant change, showing stronger resistance to abiotic stress, especially under cold stress and salt stress conditions. Resistance was significantly improved by the positive regulation of related resistance genes (such as *AtCBF1*, *AtCBF3*, *AtCOR15a*, *AtRD29a*, *AtNHX1*, *AtSOS1*, *AtSOS2*, *AtSOS3*). These results indicate that overexpression of *VhMYB60* can effectively enhance plant tolerance to cold and salt stress, and provide a valuable molecular basis for high-quality breeding of grape rootstocks.

## Data Availability

Data is contained within the article and Supplementary Materials.

## Supplementary Materials

The following supporting information can be downloaded at: https://www.mdpi.com/article/10.3390/ijms26041695/s1.
