# Supplementary material for: Arabidopsis thaliana Plants’ Overexpression of the MYB Transcription Factor VhMYB60 in the Face of Stress Hazards Enhances Salt and Cold Tolerance
Source: Int J Mol Sci. 2025 Feb 17;26(4):1695. doi: 10.3390/ijms26041695 (PMC11855753; doi:10.3390/ijms26041695)
Supplement: Supplementary file 1 [file ijms-26-01695-s001.zip › VhMYB60(Table S1) .pdf]

Table S1. List of primers used in this study.

Primer Name	Primer Sequence (5'→3')	Purpose
VhMYB60-F	ATGGGAAGGCCTCCTTGCTGTGATA	full-length cDNA of *VhMYB60*
VhMYB60-R	TCAGAATATTGGAGAGAGTTGATCC	full-length cDNA of *VhMYB60*
HR-pcr-F	GACGTCTTCGAGCTCGGTACCATGGGAAGGCCTCCTTGCT	PCR for homologous recombination
HR-pcr-R	CATGTCGACTCTAGAGGATCCGAATATTGGAGAGAGTTGATCCATCA	PCR for homologous recombination
VhMYB60-qF	CATACCTCCCTCAAAGAACTG	qPCR
VhMYB60-qR	TTCTTCCAAGCTGCTGTTTTGG	qPCR
PfActin-F	AATGGAATCTGCTGGAAT	qPCR
PfActin-R	ACTGAGGACAATGTTACC	qPCR
AtNHX1-F	AGCCTTCAGGGAACCACAAT	qPCR
AtNHX1-R	CTCCAAAGACGGGTCGCATG	qPCR
AtSOS1-F	TTCATCATCCTCACAATGGCTCTAA	qPCR
AtSOS1-R	CCCTCATCAAGCATCTCCCAGTA	qPCR
AtSOS2-F	GCAAGGGAAGAAGAAGAAGT	qPCR
AtSOS2-R	TCTCCGCTACATAACTGCC	qPCR
AtSOS3-F	GAATCCATCGCTCATCAA	qPCR
AtSOS3-R	CCATTTCTTCCTCTTCACA	qPCR
AtCBF1-F	TCGGGACTTTCCAAACCG	qPCR
AtCBF1-R	CCATCTCCTTCGCCGTCAT	qPCR
AtCBF3-F	TCCGGTAAGTGGGTTTGTGAG	qPCR
AtCBF3-R	AACTCGGCATCTCAAACATCG	qPCR
AtCOR15a-F	CAACAGAGGAATCACCAGCGA	qPCR
AtCOR15a-R	CTCTGCTGTCTTGTCGTGGTGT	qPCR
AtRD29a-F	CAACGAGGGGAAGATAAAAGTGT	qPCR
AtRD29a-R	AGCCAGATGATTTTGGAGCCT	qPCR
AtActin-F	CCCGCTATGTATGTCGC	qPCR
AtActin-R	AAGGTCAAGACGGAGGAT	qPCR
