# Supplementary material for: Arabidopsis thaliana Plants’ Overexpression of the MYB Transcription Factor VhMYB60 in the Face of Stress Hazards Enhances Salt and Cold Tolerance
Source: Int J Mol Sci. 2025 Feb 17;26(4):1695. doi: 10.3390/ijms26041695 (PMC11855753; doi:10.3390/ijms26041695)
Supplement: Supplementary file 1 [file ijms-3413478-Figure S1, Figure S2.pdf › ijms-3413478-Figure S1, Figure S2.pdf]

```
1    ATGGGAAGGCCTCCTTGCTGTGATAAAGTTGGTATCAAGAAGGGTCCTTGGACCCCAGAAGAGGACATCATCTTGGTCTCCTATATCCAA
1     M  G  R  P  P  C  C  D  K  V  G  I  K  K  G  P  W  T  P  E  E  D  I  I  L  V  S  Y  I  Q

91   GAGCATGGCCCCGGAAATTGGAGATCAGTGCCTACAAACACCGGGCTGCTGAGGTGTAGCAAGAGTTGCAGGCTTAGATGGACTAATTAC
31    E  H  G  P  G  N  W  R  S  V  P  T  N  T  G  L  L  R  C  S  K  S  C  R  L  R  W  T  N  Y

181  CTTAGACCGGGGATTAAGCGCGGTAACTTCACTCCCCATGAAGAAGGAATGATCATCCATCTACAAGCCCTATTGGGTAACAAATGGGCT
61    L  R  P  G  I  K  R  G  N  F  T  P  H  E  E  G  M  I  I  H  L  Q  A  L  L  G  N  K  W  A

271  GCCATAGCTTCATACCTCCCTCAAAGAACTGATAATGATATAAAGAATTATTGGAACACTCACTTGAAGAAGAAGATCAAGAAGTTTCAG
91    A  I  A  S  Y  L  P  Q  R  T  D  N  D  I  K  N  Y  W  N  T  H  L  K  K  K  I  K  K  F  Q

361  TCAGCTTTGAGCCCCCATATGGCCTCAGATTCAACCACAAGCACCTGTACTAATCATCAATTTGTACCAAGGAGCTATGCCGGTGATGAT
121   S  A  L  S  P  H  M  A  S  D  S  T  T  S  T  C  T  N  H  Q  F  V  P  R  S  Y  A  G  D  D

451  CATCACAGAAGGGGTAGTTCTTTTGAGGTCATCAATGGCCATTCCTCGGCTCACCCGAGCCTAAACAGCCCGATCTCGACGTATGCCTCC
151   H  H  R  R  G  S  S  F  E  V  I  N  G  H  S  S  A  H  P  S  L  N  S  P  I  S  T  Y  A  S

541  AGCACTGAGAACATCTCAAGGCTACTAGAAGGTTGGATGAGGTCCTCCCCAAAGGCCACCAAGGAGAAACTGTACCAAAACAGCAGCTTG
181   S  T  E  N  I  S  R  L  L  E  G  W  M  R  S  S  P  K  A  T  K  E  K  L  Y  Q  N  S  S  L

631  GAAGAAGGTAGTATCGATATGACCGGAAACTCCATGGCGGTGGCGGCGGTGACTTCTGTCCAGTGTTACAGGCCTAAGTTGGAGCAAGGA
211   E  E  G  S  I  D  M  T  G  N  S  M  A  V  A  A  V  T  S  V  Q  C  Y  R  P  K  L  E  Q  G

721  GGGGGCGAGTTGGTCGCCAACGACGAGTTGGAGTCCATTCTTGAGTACGAAAACCTGAATGATGATCATCATCAGACTACTGATACTACT
241   G  G  E  L  V  A  N  D  E  L  E  S  I  L  E  Y  E  N  L  N  D  D  H  H  Q  T  T  D  T  T

811  ATTCCAAGTGATGATCATGATCATGTTCATGATCATGAGATGAAGATGGATCATGATCAGAAGAAGCACAACCCTCCTCTATCATTTCTT
271   I  P  S  D  D  H  D  H  V  H  D  H  E  M  K  M  D  H  D  Q  K  K  H  N  P  P  L  S  F  L

901  GAGAAATGGCTCTTGGATGAATCAGCAGCTCAAGGAGAGGAGATGATGGATCAACTCTCTCCAATATTCTGA
301   E  K  W  L  L  D  E  S  A  A  Q  G  E  E  M  M  D  Q  L  S  P  I  F  *
```

Supplement Figure S1. Nucleotide and amino acid sequence of *VhMYB60*. The parts underlined in green and orange are the conserved domains of r repeats.

A

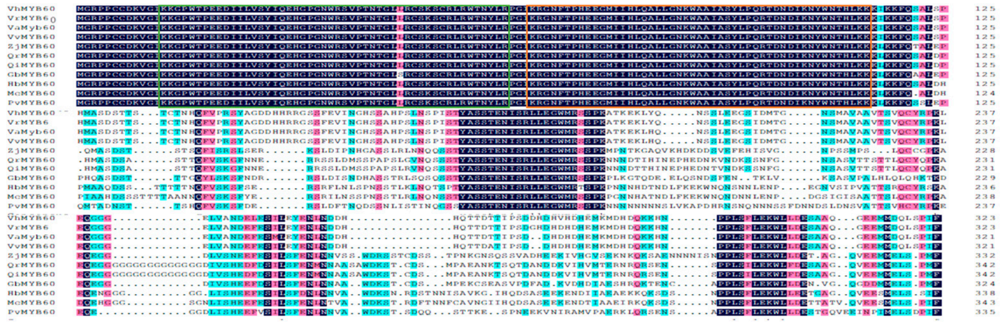

B

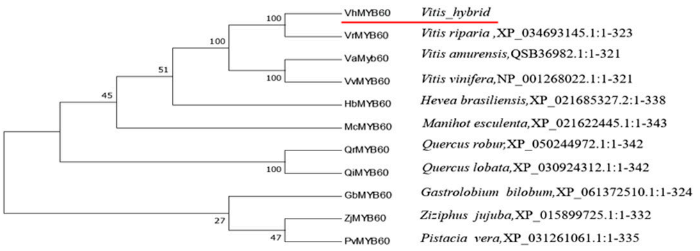

Supplement Figure S2. Multiple sequencing analysis of *VhMYB60* protein. (A) MYB sequence alignment. Evolutionary tree (B). Target proteins are represented by lines, while R-repeat structures are represented by green and orange boxes.
